# Supplementary material for: A Global Screen for Assembly State Changes of the Mitotic Proteome by SEC-SWATH-MS
Source: Cell Syst. 2020 Feb 26;10(2):133–155.e6. doi: 10.1016/j.cels.2020.01.001 (PMC7042714; doi:10.1016/j.cels.2020.01.001)

Q9BSL1 | UBAC1_HUMAN | UBAC1 GBDR1 KPC2 UBADC1
Monomer MW [kDa]: 45.338 Monomer expected elution fraction: 47

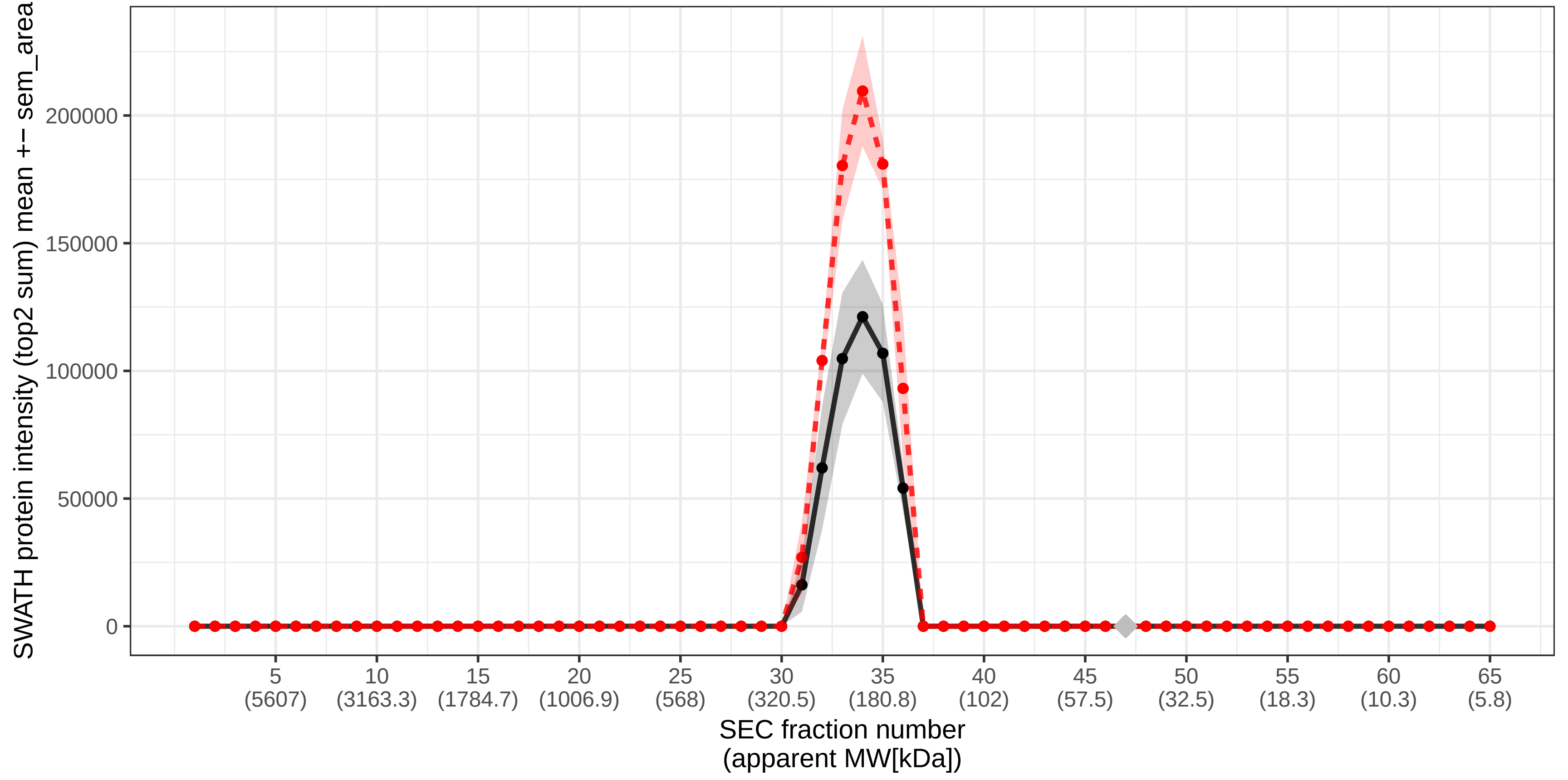

Supplement: Data S1. SEC-SWATH-MS Protein Chromatograms, Related to Figure 1 [file mmc6.zip › SECchrom_Q9BSL1_UBAC1_HUMAN_UBAC1_GBDR1_KPC2_UBADC1.pdf]
